# *Fmr1* KO Mice as a Possible Model of Autistic Features

**DOI:** 10.1100/tsw.2006.220

**Published:** 2006-09-20

**Authors:** Maude Bernardet, Wim E. Crusio

**Affiliations:** Laboratoire de Neurosciences Cognitives, CNRS UMR 5106, Université de Bordeaux I, Bat B2 - Avenue des Facultés, 33405 Talence Cedex, France

**Keywords:** animal model, autism, autistic-like traits, behavior

## Abstract

Autism is a pervasive developmental disorder appearing before the age of 3, where communication and social interactions are impaired. It also entails stereotypic behavior or restricted interests. Although this disorder was first described in 1943, little is still known about its etiology and that of related developmental disorders. Work with human patients has provided many data on neuropathological and cognitive symptoms, but our understanding of the functional defects at the cellular level and how they come about remains sketchy. To improve this situation, autism research is in need of valid animal models. However, despite a strong hereditary component, attempts to identify genes have generally failed, suggesting that many different genes are involved. As a high proportion of patients suffering from the Fragile X Syndrome show many autistic symptoms, a mouse model of this disorder could potentially also serve as a model for autism. The *Fmr1* KO mouse is a valid model of the Fragile X Syndrome and many data on behavioral and sensory-motor characteristics of this model have been gathered. We present here an assessment of autistic features in this candidate model. We conclude that *Fmr1* KO mice display several autistic-like features, but more work is needed to validate this model.

## AN OVERVIEW OF AUTISM

Autism is classified as a developmental pervasive disorder, the diagnosis of which is based on behavioral symptoms and age of onset[1], with the earliest manifestations appearing before the age of 3. Essential behavioral features of autistic disorder fall into three classes: abnormal or altered social interactions, abnormal or altered social communication, and a restrained repertory of interests and activities. Symptoms vary greatly between individuals[2,3,4]. Due to the variable clinical picture and the accompanying blurring of diagnostic classes, estimations of the prevalence of autism vary widely, but cluster around 6 births per 1000[5,6,7,8,9,10]. The syndrome is much more frequent in boys than girls, with a ratio around 4:1.

Altered nonverbal social and communicative behaviors concern social gaze, facial mimics, body postures, and gestures[1,3]. Autistic individuals often fail to establish relations with peers corresponding to their levels of development. They may not spontaneously share their interests, pleasures, or achievements with other people, and may lack social or emotional reciprocity[3,11,12]. Qualitative communication alterations include a late display or complete lack of spoken language development without compensation by nonverbal communication[1]. Even in subjects who master language sufficiently, an inability to engage or sustain a conversation with a peer can be observed. Stereotyped and repetitive use of language or idiosyncratic speech may occur. Lack of spontaneous playacting and of social imitation play according to age of development also occurs frequently. The restrictive, repetitive, and stereotyped character of behaviors, interests, and activities may be manifested by a preoccupation circumscribed to one or more interest centers that is abnormal in its intensity or in its orientation. This may also show as inflexible adhesion to habits, unspecific and nonfunctional rituals, or by stereotyped motor mannerisms or persistent preoccupation for objects or parts of the body[3,13].

The core features of autism described above, defining the diagnosis of this disorder, are also frequently associated with variable accompanying symptoms[1]. In most cases, mental retardation occurs, the severity of which varies from light to profound (see [14] for a discussion). The profile of cognitive capacities is usually heterogeneous and some particular skills may occur at a much higher level than most other skills. Individuals with autism can display a variety of behavioral traits, such as hyperactivity, attention deficit[15], impulsivity, self-injurious behavior, and, particularly in the youngest, anger crises[16]. Mood or affect perturbations are frequent[17], as are disturbed sleep patterns[18,19]. The child can lack fear in some dangerous situations, but show excessive fear in others. Responses to sensory stimuli can be abnormal, e.g., a high threshold to pain, and hypersensitivity to noise and physical contact, overreaction to lights or odors, or fascination for certain stimuli[20]. Convulsions occur in 5–38% of cases, particularly before 5 years of age or in teenagers[21].

## THE PUZZLE OF AUTISM: WHAT WE KNOW AND HOW WE KNOW IT

Since its description in 1943 by Kanner[3], there have been many efforts to link the behavioral features of autism to underlying neural abnormalities. Psychiatric observations, combined with data from experimental psychology, autopsies, and anatomic and functional magnetic resonance imaging, have started to shed some light on the nature of this affliction. The anomalies observed in different brain structures have led to different hypotheses implicating dysfunctions of the amygdala[11], orbitofrontal-amygdala circuit[22], frontal-striatal system, and cerebellum[23]. In addition, alterations in various neurotransmitter systems have been postulated[2].

Many structures were found to have an altered cytoarchitecture in autistic patients' brains: corpus callosum[24,25,26], parts of the limbic system, cortex[27,28], cerebellum, and brainstem, but observations were not always replicated. In the limbic system, the hippocampus, amygdala, entorhinal cortex, subiculum, mammillary body, anterior cingulate cortex, and septum display small cell sizes and increased cell packing densities at all ages (reviewed in [29,30]). Golgi analysis of CA1 and CA4 pyramidal neurons has shown decreased complexity and extent of dendritic arbors in these cells[31]. The cerebellum is also affected. Decreases in numbers of Purkinje cells were systematically observed, particularly in the inferior posterior hemisphere regions of the cerebellum[27,32,33,34].

Alterations may also differ as a function of age. Studies suggest that neurons in the vertical limb of the diagonal band of Broca, in cerebellar nuclei, and in the inferior olive are abnormally large and numerous in young individuals with autism, but are small, pale, and significantly reduced in number in adult autistic individuals[27,29,30]. The events resulting in the observed decreased numbers of Purkinje cells may occur before the connections between fibers of the olivary neurons and the Purkinje cells are formed, and may account for a possible prenatal cause of autism (see [30]).

It was found that brains of children with autism are larger than those of age-matched controls, while the brains of autistic adults tend to be lighter than controls[35]. Brain enlargement seems to be postnatal as newborns show few or no differences with controls[36]. Many mechanisms have been hypothesized to explain these observations, such as increase in neurogenesis, decrease in neuronal apoptosis, increase in glial cell production, diminished synaptic pruning, or myelin abnormalities, but none has been confirmed.

It has been proposed that autistic disorders may have viral[37], autoimmune[38], teratogenic[39], or genetic origins[40,41], none of these hypotheses being exclusive of the others. Congenital and neonatal TORCH infections (acronym for Toxoplasmosis, Others [syphilis, varicella-zoster, and parvovirus B19], Rubella, Cytomegalovirus, and Herpes) or autoimmune reactions to these pathogens have been associated with autism[42,43]. Drug use during pregnancy has also been linked to increased frequency of autism. For example, anticonvulsants, the mood stabilizer valproic acid, and antiemetic thalidomide have teratogenic effects during early stages of intrauterine development. Minor malformations that occur frequently in people with autism are known to arise in the same stages[39].

Support for a genetic basis of autism comes from a variety of sources, such as epidemiologic surveys, family and twin studies, and linkage analyses[41,44,45]. Gross disruptions of chromosomal material account for about 5% of cases of autism[46,47,48] and 5–12% of cases arise from disorders that affect the brain and have a known genetic etiology, such as tuberous sclerosis and neurofibromatosis[49,50]. Samples of the remaining cases of idiopathic autism have been examined for heritability in family and twin studies. Around 5% of siblings of autistic individuals will also develop the disorder; a rate that is 50 times higher than the 0.1% prevalence of autism in the general population[6,51]. In addition, 60–90% of monozygotic twins are concordant for this disorder[52,53,54] and many family members of probands appear to present various autistic traits, but in a milder form[55]. Genetic linkage studies have been conducted on autistic probands and their families. Numerous loci have been found to be potentially linked to the disorder, most of them being more or less specific for a given population[44]. Candidate genes include alleles of genes implicated in development, e.g., Reelin and engrailed2, and others, such as the SERT gene (serotonin transporter; [56,57]).

In conclusion, studies on humans have increased our understanding of autism and its developmental origin is now generally accepted. Nevertheless, we still lack specific hypotheses to explain not only autistic symptoms, but also their heterogeneity and their emergence during development. Human studies are limited due to the scarcity of study material, personal and uncontrollable history of individuals, variability and uncontrollability of genetic background, inability to isolate genetic and environmental factors, indirect inference of brain operation within the limitations of current noninvasive methods to investigate the brain, and the difficulty with which experimental results replicate. A valid animal model, therefore, could clearly help to advance our knowledge significantly. A major challenge of any model of autistic brain development is to take into account the neural substrates implicated and the variations that can be observed between affected individuals.

## FURTHER UNDERSTANDING: LOOKING FOR ANIMAL MODELS

It will be evident that no exact mouse equivalent can exist for such exquisitely human traits as are affected in autism, or for any of the other common psychiatric disorders, for that matter. Moreover, some human brain structures hardly have an equivalent or develop differently in mice. The prefrontal cortex, for example, is thought to be involved in cognitive rigidity and is poorly developed in mice[58]. In order to be useful, however, an animal model does not need to recapitulate a human disorder or syndrome exactly. Indeed, it would appear that several of the intermediate traits (or endophenotypes) of autism can be modeled in animals[59,60].

For an animal model to be considered relevant in psychiatric research, it should meet different criteria of validity, such as construct, face, and predictive validity[61,62,63]. Thus, an acceptable animal model for autism should reflect the developmental problems discussed above to satisfy the criterion of construct validity. Face validity means that the model should display autistic-like behavioral traits resembling core symptoms of autism concerning social relations, social communication, and restricted activities, and should at least approximate most of the variable symptoms, such as anxiety, mental retardation, clumsiness, aggression, hyperactivity, abnormal sensory responses, and stereotypies. Several behavioral tasks have been designed for mice to assess autistic-like traits[64]. If indeed present, such behavioral traits could be studied in the model after challenge with possible medications used in autistic patients to improve particular symptoms. Predictive validity of the model would be established when these drugs reduce or improve symptoms not only in the model, but in human patients, too.

Primates have been used to study social relations and social communication, but they have limitations because of their high financial cost, long developmental time span, the fact that genetic manipulation is impractical, and ethical considerations. Rodents do not suffer from these drawbacks. A limitation to the use of rodent models for autism is the difficulty to model specific human features such as social gaze and sharing of interests. Nevertheless, as we will see below, most behavioral features of autism can be modeled in mice, which therefore could render valuable models for autism.

Belzung et al.[58] classified models for autism in four categories: (1) animals mutated for neuropeptides implicated in social behavior and attachment (vasopressin, oxytocin, µ-opioid receptors), (2) models of epigenetic factors implied in autism (developmental deficits in serotonin, fetal exposition to anticonvulsants or thalidomide), (3) neonatal lesions of autism-associated structures, and (4) models of genetic diseases associated with autism (e.g., Fragile X or Rett Syndrome and others). The problem with the majority of these models is that they concern only some aspects of the etiology and not the whole symptomatology, although the functional role of several structures has become better understood with their help. Moreover, most of these models have up till now only been tested for a few autistic-like traits. In what follows, we will review evidence that one of the models of Belzung's fourth category, the *Fmr1* KO mouse, might also serve as a model for autism.

## THE FRAGILE X SYNDROME

The *FMR1* mutation underlying the Fragile X syndrome (FXS) is the most frequent cause of inherited mental retardation and is interesting here because about 10–30% of these patients are also diagnosed with autism[49,65,66,67]. FXS accounts for around 5% of the autistic population[49,68,69]. Many autistic behavioral traits are common in FXS, even in patients that are not formally diagnosed with autism. FXS also shares many of the variable features of autism such as hyperactivity[70], stereotypical behavior, aggressiveness, anxiety (particularly due to social stress[70]), disturbed sleep patterns[71], high prevalence of epilepsy[72], and impairment in sensorimotor gating[73]. Both disorders are developmental and affect more boys than girls. It is interesting to note that although due to a single mutation, FXS symptoms are very variable in quality and severity between individuals, another parallel with autism. For all these reasons, autism researchers have become increasingly interested in FXS[74].

The FMRP protein, the product of the *FMR1* gene, is expressed in brain tissue and in testes. In neurons, it is localized either in the nucleus or cytoplasm[75,76,77], depending on the splicing[75]. It complexes with other proteins[78] and is implicated in mRNA transport and translation regulation[78,79,80,81]. Its own translation is thought to be dependent on synaptic activity. Although much is known about its conformation, expression pattern, and localization, its role in mental retardation remains to be elucidated.

A knock-out mutation has been induced in the mouse *Fmr1* gene, which is 98% similar to its human ortholog, *FMR1*[82]. FMRP expression was disrupted by introducing a neomycin cassette into exon 5 of the gene[82]. Recently, Yan et al.[83] observed some residual *Fmr1* RNA expression in these animals that may be due to alternative splice variants. Nevertheless, *Fmr1* KO mice display macroorchidism and cognitive and other behavioral deficits comparable to those of human FXS patients[82]. In addition, the abnormally long and thin dendritic spines characteristic of FXS patients are also found in young KOs[84,85,86,87,88]. *Fmr1* KO mice have been tested for numerous behavioral tasks relevant to FXS and these animals have therefore been validated as a model for this disorder[89]. The relatively lighter symptomatology of the disorder that has been observed in these mice for some features may be due to the above-mentioned residual expression of splice variants of *Fmr1* RNA[83].

The *Fmr1* KO was produced in a 129 ES cell line and the resulting mutants were then recurrently backcrossed to both C57BL/6J (B6) and FVB/N (FVB) animals. It appears that the mutation expresses differently on these two backgrounds[90,91].

## *Fmr1* KO MICE: BEHAVIORAL CHECKUP RELEVANT TO AUTISTIC TRAITS

*Fmr1* KO mice have been extensively studied for a large variety of behavioral and sensorimotor traits (see Tables 1 and 2). Most studies aimed to validate these mice as a model for FXS and thus only relatively few studies have assessed behavior relevant to the core symptoms of autism. An exception is abnormal social behavior, which is also a prominent feature of FXS.

**TABLE 1**
**Phenotypical Checkup of *Fmr1* KO Mice: Behaviors Relevant to Core Symptoms of Autism**

| Test | Background | Result | Ref. |
|---|---|---|---|
| **Inappropriate social interactions** | | | |
| Mirrored chamber test | B6 | KO < WT for % time in the mirrored chamber | [94] |
| Tube test of social dominance | B6 | KO < WT vs. unfamiliar WT the first time<br>KO = WT vs. unfamiliar WT the third day<br>KO = WT vs. familiar WT | [94] |
| Social interaction test | B6 | KO vs. WT:<br>Active social behavior: KO > WT<br>Passive social behavior: KO < WT<br>KO vs. KO, WT vs. WT:<br>Sniffing and receptive behavior: KO > WT | [94] |
| | | KO vs. C3H, WT vs. C3H: KO < WT | [95] |
| Crawley test | B6 | KO = WT | [94] |
| Influence of cage familiarity on response to unfamiliar social partners | B6 | In an unfamiliar cage: KO = WT; in a familiar cage: KO < WT during the first 5 min, KO > WT after 20 min | [94] |
| **Perseverance** | | | |
| Water maze reversal learning: | | | |
| Hidden-platform condition | B6 | KO = WT | [97,98] |
| | B6 | Escape latencies: KO > WT | [82,89,96] |
| | B6 | Path length: KO > WT | [96] |
| | B6 | Number of trials: KO > WT | [98] |
| | B6 | Rate of learning: | |
| | | KO = WT, | [96] |
| | | KO>WT | [89] |
| Visible-platform condition | B6 | Escape latencies: | |
| | | KO > WT | [96] |
| | | KO = WT | [82] |
| E-shaped water maze reversal learning | B6 | KO = WT | [89] |
| Plus-shaped water maze reversal learning | B6 | Escape latencies: KO = WT, but rate of learning: KO < WT | [98] |

Although some core symptoms, such as sharing pleasures and interests with others, can hardly be modeled in rodents, the inability to establish normal relations with peers and the lack of social reciprocity can be assessed more easily. The mirrored chamber test, for instance, was developed by Seale and his team[92] based on the notion that most animals react to their mirror image as if it was another animal[93]. The apparatus consists of a box, the interior of which is totally mirrored, laid out in the center of a dark open field; the wall opposite the chamber is also mirrored. *Fmr1* KO mice were found to spend less time in the center mirrored chamber compared to total time spent in the mirrored alley (considered to be less anxiogenic) and the mirrored chamber[94]. A tube test can be used to evaluate social dominance. If two

**TABLE 2**
**Phenotypical Checkup of *Fmr1* KO Mice: Behaviors Relevant to Variable Symptoms of Autism**

| Test | Background | Result | Ref. |
|---|---|---|---|
| **Anxiety** | | | |
| Elevated plus maze | FVB | KO = WT | [100] |
| | B6 | KO = WT | [91,107] |
| | FVBxB6 | KO = WT | [107] |
| | FVBxB6 | KO less anxious than WT | [83] |
| Thigmotaxis in open-field | B6 | KO < WT | [94,101] |
| | FVBxB6 | KO < WT | [83] |
| Boli in open-field | B6 | KO < WT | [94] |
| Light-dark exploration | B6 | Transitions between compartments: KO > WT<br>Time spent in both compartments: KO = WT | [82,101] |
| Corticosterone response to acute stress | B6 | Males:<br>Sham and 15 min: KO = WT<br>0 min: KO < WT<br>60 min: KO > WT<br>Females:<br>Sham, 0 and 60 min: KO = WT<br>15 min: KO < WT | [104] |
| | B6 | Males:<br>No stress, 30 min stress: KO = WT<br>2 h stress: KO > WT | [103] |
| Conditioned emotional response | B6 | KO = WT | [98] |
| **Learning and memory** | | | |
| Cross-shaped water maze | FVB | Correct trials: KO < WT | [102] |
| | B6 | Escape latencies: KO = WT | [98] |
| | | Correct trials: | |
| | | KO < WT | [98] |
| | | KO = WT | [102] |
| Changing position of platform in water maze | B6 | KO = WT | [97,98] |
| E-shaped water maze | B6 | KO = WT | [89] |
| Morris water maze training: | | | |
| Hidden-platform condition | B6 | Escape latencies: | |
| | | KO = WT | [96,97,101] |
| | | KO > WT | [89] |
| | | KO > WT the first four trials | [82] |
| | FVBxB6 | Escape latencies: KO > WT | [83] |
| | B6 | Rate of learning: KO = WT | [82,89,102] |
| | FVB | Rate of learning: KO < WT | [102] |
| Visible-platform condition | B6 | Escape latencies: KO = WT | [82,96] |
| Radial maze | B6 | Working memory: KO = WT | [91] |
| | FVBxB6 | Working memory: KO < WT the first 6 days; reference memory: KO < WT; strong choice design: KO = WT | [83] |
| Barnes maze | FVBxB6 | KO = WT; during probe test: KO < WT | [83] |
| Fear conditioning: context and conditioned cue | FVB | KO = WT | [102] |
| | B6 | KO = WT | [98,101,102] |
| | B6 | KO < WT | [97] |
| Trace fear conditioning | B6 | KO < WT | [100] |
| Conditioned eyelid blink reflex | B6 | KO < WT | [109] |

**TABLE 2 (continued)**

| Test | Background | Result | Ref. |
|---|---|---|---|
| **Learning and memory (continued)** | | | |
| Passive avoidance (latency to enter dark compartment) | B6 | KO = WT | [82] |
| | FVB | KO = WT | [108] |
| Lever press escape/avoidance task | B6 | KO < WT | [113] |
| Instrumental conditioning | B6 | Conditioning learning : KO = WT | [73] |
| | | Devaluation of reward and omission of lever press : KO > WT | |
| Olfactory learning and memory tasks | FVBxB6 | KO = WT | [83] |
| Novel object task | FVBxB6 | KO = WT | [83] |
| | FVB | KO < WT | [114] |
| **Motor abilities** | | | |
| Rotarod motor coordination and balance | B6 | KO = WT | [101] |
| **Aggression** | | | |
| Neutral cage aggression test | B6 | KO = WT | [91] |
| **Hyperactivity** | | | |
| Open field activity | B6 | KO > WT | [91,94,101] |
| | B6 | KO = WT | |
| | FVBxB6 | KO = WT | [107] |
| | FVB | KO = WT | [107] |
| | FVB | KO = WT before 18 min | [100] |
| | | KO > WT after 18 min | [108] |
| Activity cage | FVB | KO > WT | [114] |
| Motor activity test | B6 | KO > WT | [82] |
| **Idiosyncratic responses to sensory stimuli** | | | |
| Auditory startle response | B6 | KO = WT, but increased response with Fmr1gene containing YAC | [101] |
| | B6 | KO > WT at 70 and 80 dB; KO < WT at 120 dB | [107] |
| | B6 | KO < WT at higher intensities, interaction between genotype and intensity | [73] |
| | FVB | KO < WT | [110] |
| | FVB | KO = WT under 110 dB; KO< WT from 110 dB and above | [108] |
| | FVBxB6 | KO > WT at 80 dB; KO < WT at 100, 110, and 120 dB | [83] |
| | FVBxB6 | KO = WT | [83] |
| Prepulse inhibition | B6 | KO > WT | [73] |
| | B6 | KO > WT at 67 dB (2 dB above background noise) | [107] |
| | FVB | KO > WT | [110] |
| Audiogenic seizures (AS) | FVB | KO after long loud sound and after age 10 weeks | [110] |
| | | KO >> WT (143 ± 5 days) | [115] |
| | | KO >> WT (45 days and under) | [108] |
| | B6 and FVBxB6 | KO display AS, WT do not (21 days) | [83] |
| | FVB | KO >> WT (30 days) | [83] |
| Hot plate and tail-flick test | FVB | KO = WT | [100] |

mice enter a tube at opposite sides, meeting in the center, generally, one will push back the other and hence be called dominant. The number of matches won by KO mice appeared to be dependent on the familiarity of the opponent animal. Familiarity of the environment also has an effect on social interactions; KO and WT spent as much time at the interface of two compartments with an unfamiliar mouse in an unfamiliar cage, but they displayed a different pattern of social exploration when put in a familiar cage[94].

Social interaction tests conducted by Spencer et al.[94] revealed that KO mice showed increased active social behaviors when confronted with a WT or KO animal, increased receptive social behaviors when opposed to a WT, and decreased receptive social behaviors when confronted with KO peers. In the social interaction test conducted by Mineur et al.[95] using C3H ovariectomized females as stimulus mice, KO displayed decreased social behavior in comparison to WT. A partition test (noncontact version of the social interaction test) revealed no difference between KO and WT[94]. Despite some divergent results most probably due to procedural differences, both groups concluded that social behavior is abnormal in KO compared to WT.

A second group of core symptoms of autism concerns repetitive and stereotypic behaviors, resistance to change, and restricted activities. To date, perseverance is the only aspect that has been investigated, mostly on a B6 genetic background. It has been studied by the rate of extinction following training to swim to a platform in various types of water mazes. Three of the studies found that *Fmr1* KO mice had significantly longer latencies to reach the changed position of a platform after learning an initial position[82,89,96], whereas two other studies did not find any differences with WT[97,98]. Van Dam and colleagues[98] used a cross-shaped water maze and found that although escape latencies were similar to WT, *Fmr1* KO mice made significantly less correct trials. These results seem to indicate that KO animals are less flexible than WT and tend to persist in a once learned habit longer.

Regarding the variable symptoms of autism, most tests conducted on *Fmr1* KO mice concerned the murine equivalents of anxiety, mental retardation, hyperactivity, and idiosyncratic responses to sensory stimuli (Table 2). Some features were found to be altered, but some showed no differences. Anxiety is very often observed in autistic people and is linked to the impairment in anticipation that is thought to be the origin of ritualistic behaviors[3]. This feature could also possibly be due to an alteration of amygdala function[99]. An amygdala defect seems indeed to be present in *Fmr1* KO mice as indicated by a trace fear conditioning deficit[100] and altered social interactions. Most classical anxiety tests did not show any differences between KO and WT[98,100,101,102], however, or KO were found to be even less anxious than WT[82,91,94,101]. Abnormalities in corticosterone levels in response to restrain stress have nevertheless been shown[103,104], resulting in altered negative feedback regulation of the glucocorticoid response. In consequence, *Fmr1* KO mice present a delayed response to stress and are also slower to return to baseline[104]. FMRP binds to the glucocorticoid receptors and their expression was reduced in the dendritic region of the *Fmr1* KO mouse hippocampus[105]. It has indeed been reported that FXS patients have a deregulated adrenopituitary axis[106].

Some studies on learning capacities indicated a deficit of KO mice in spatial learning tasks compared to WT mice[82,83,98,102] while others did not report any deficit[83,91,97,101]. Deficits were found in trace fear conditioning and were linked to LTP deficits in the lateral amygdala and cingulate cortex[100]. Similar deficits were found in *Fmr1* KO on a mixed 129/FVB hybrid genetic background[102], which were investigated in only this one study.

Motor abilities and aggression have been reported to be normal[91,101], but this may need further exploration. Hyperactivity, however, seems to be the most consistent behavioral feature of *Fmr1* KO mice on a B6 genetic background[82,91,94,101] although Nielsen et al.[107] reported no differences after a 5-min observation in the open field. Hyperactivity in *Fmr1* KO on a FVB/N background is still controversial as they were reported not to be different from control mice[100] (as was the case in KO on an FVBxB6 hybrid background[107]), but were significantly increased when test durations were longer than 18 min[108].

Although no difference was found between KO and WT in nociception in response to heat, most other responses to sensory stimuli were found to be altered in KO[100]. Koekkoek et al.[109] reported that conditioned eye blink reflex was altered in a Purkinje cell specific *Fmr1* KO on a B6 background. Auditory startle response also seems to be FMRP dependent; although KO on both FVB and B6 backgrounds displayed opposite responses, they were both significantly different from WT[73,101,107]. Finally, *Fmr1* KO mice on both genetic backgrounds were radically more prone to audiogenic seizures than their WT homologues[83,110].

## CONCLUSIONS

The *Fmr1* KO mouse model has been studied for several behavioral and physiological features relevant to autism and appears to display most expected symptoms. Although mixed results have been obtained for a few behavioral features, other autism-specific features are undoubtedly impaired. Most results up till now have been obtained with KO animals on the B6 background. It should be noted that in spite of the relatively scarce data, it is clear that FVB mice also are affected by the KO mutation of *Fmr1,* but present a specific behavioral profile different from the one displayed by B6 KOs. In particular, the *Fmr1* KO mutation was shown to have opposite effects on the sizes of the hippocampal intra- and infrapyramidal mossy fiber (IIPMF) terminal fields, depending on whether the mutation was expressed on a B6 or FVB background[90,91]. These IIPMF seem to be implicated in several behaviors[111,112]. Thus, it should be interesting to carry out more systematic studies in both the B6 and FVB backgrounds. This might render a plastic and reliable model for autism that takes into account the variability of the disorder in humans.

Despite the foregoing, it is clear that much work remains to be done before *Fmr1* KO mice can be validated as a model for autism. In particular, social communication and cognitive rigidity have hardly been investigated yet. Several variable features of autism, such as sleep disturbance, brain overgrowth, developmental delays, and stereotypies, also need more detailed investigation.

In conclusion, the etiology of autism remains unknown and the limitations of research in humans make it necessary to develop animal models for this disorder. These models must fit construct, face, and predictive validity criteria. Several possible models exist, but few of them have been extensively studied as yet. The current short review shows that not only does the Fragile X Syndrome have a symptomatology resembling autism to a very large extent and that the validated genetic mouse model that is available for this disorder, the *Fmr1* KO mouse also shows much promise as a possible model for autism.

## ACKNOWLEDGMENTS

Supported by grants from the March of Dimes (12-FY05-1198), Conseil Régional d'Aquitaine, CNRS, and the University of Bordeaux I to WEC.
